# Modeling the Growth of *Listeria monocytogenes* Isolates in Low‐Temperature Environments

**DOI:** 10.1002/fsn3.70987

**Published:** 2025-09-19

**Authors:** Yue Cheng, Peter Myintzaw, Francis Butler

**Affiliations:** ^1^ UCD School of Biosystems and Food Engineering University College Dublin Dublin Ireland; ^2^ Department of Biological Sciences Munster Technological University Cork Ireland

**Keywords:** growth models, *Listeria monocytogenes*, low‐temperature growth

## Abstract

The research explored the growth dynamics of *Listeria monocytogenes* at 4°C and 7°C using five microbial growth models, identifying significant variability in growth rates among isolates. The Spline model exhibited greater variability and less reliability, leading to the selection of average growth rates from the other four models for further analysis. The study found that isolates from seafood environments exhibited higher growth rates at both temperatures compared to those from meat and mixed food environments. Moreover, the growth rate of isolates from vegetables increased more rapidly when the temperature was raised from 4°C to 7°C, compared to isolates from clinical settings, highlighting the influence of environmental factors on the growth behavior of *L. monocytogenes* in cold environments.

## Graphical abstract

Schematic representation of the modelling the growth of Listeria monocytogenes isolates in low temperature environments.

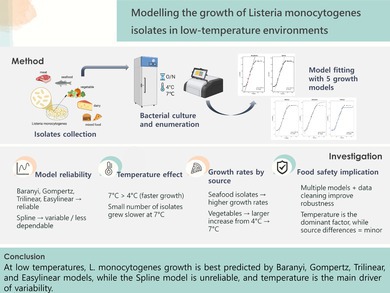

## 1 Introduction

*Listeria monocytogenes* is a Gram‐positive bacterium capable of growing in low‐temperature environments. The growth temperature range of *L. monocytogenes* is broad, spanning from 0°C to 45°C (Jones and D'Orazio 2013). Although its maximum growth rate is slower at low temperatures such as 4°C, it can still survive and proliferate. During cold chain transportation and storage, food is typically kept at low temperatures to extend shelf life and inhibit microbial growth. *L. monocytogenes* can be found in various foods, especially those that are ready‐to‐eat and not subjected to further heat treatment by the consumer. Commonly contaminated foods include dairy products (such as soft cheeses), meat products (such as hot dogs and deli meats), seafood, vegetables, and salads (Ricci et al. 2018). With the possibility of *L. monocytogenes* contamination, any temperature fluctuations or uneven cooling in the cold chain environment can lead to the proliferation of *L. monocytogenes* , thereby increasing the risk to food safety (Osek et al. 2022).

Microbial growth modeling holds significant importance in the field of food safety. By fitting growth models, it is possible to predict the behavior of microorganisms under various environmental conditions, thereby aiding in understanding the impact of factors such as temperature, pH, and nutrient availability on microbial growth (Kumar et al. 2024). This predictive capability also enables accurate estimation of food shelf life, which helps in reducing the risk of foodborne diseases. Modeling can be applied to growth data generated either by enumeration using direct plating techniques or to growth data where an indirect measurement of growth such as turbidity measurement is used (Taiwo et al. 2024). However, there is evidence (https://vcadavez.shinyapps.io/ListeriaCardinalModel/) that optical density techniques result in a higher estimate of the maximum growth rate compared to plating methods. In addition, there are several models that can be fitted to growth rate data that can result in somewhat differing estimates of the growth rate (Gonzales‐Barron et al. 2024). The objective of this study is to compare the maximum growth rates of *L. monocytogenes* at 4°C and 7°C using different growth models to fit growth data obtained using turbidity measurement.

## 2 Materials and Methods

### 2.1 *L. monocytogenes* Isolates and Growth Conditions

Maximum growth rates were established for 150 *L. monocytogenes* isolates from various food and clinical sources that were provided by Teagasc Food Research Centre, Moorepark Co Cork. Details of the isolates used are provided in Myintzaw et al. (2022). In addition, several EURL Lm reference strains of L. monocytogenes, EGD‐e, 10403S (Lauer et al. 2002), 6179 (Schmitz‐Esser et al. 2015), and F2365 (Nelson et al. 2004) were included in the study.

Experimental growth data for the 150 *L. monocytogenes* isolates at 4°C and 7°C using an optical density technique was previously established in the study by Myintzaw et al. (2022). Full details of the growth determination by optical density measurement are given in that paper. In brief, single *L. monocytogenes* colonies obtained from previously incubated BHI agar plates stored at 4°C until required were inoculated into 10 mL of BHI broth and incubated at 37°C for 18 h. The culture was then diluted with saline solution (NaCl 0.85%) to approximately 1 × 10^5^ CFU/mL. 2 μL of each diluted culture was aliquoted into wells of a 96‐well plate containing 198 μL of BHIB, in triplicate. A total plate count was used to determine the initial inoculum concentration. Reference strains *L. monocytogenes* EGD‐e and F2365 served as positive controls, and 200 μL of sterile BHIB without culture acted as a negative control. Each test plate was incubated at 4°C and 7°C. At each required time point, microtiter plates were taken out and OD600 readings were taken after 30 s of shaking using a microplate reader (MultiskanSky, Agilent, Waldbronn, Germany). Readings were taken from 0 to 430 h at approximately 16 h intervals. Three replicates were measured for each isolate at both temperatures. In addition, selected *L. monocytogenes* isolates with different maximum growth rates were cultured in BHIB at 4°C and 7°C. At designated time points, samples were serially diluted, and 10 μL of each dilution was spot‐plated on BHI agar. Plates were incubated at 30°C for 48 h, and colonies were manually counted. Each isolate was tested in three biological replicates, with duplicate plates for each dilution.

The choice of the two temperatures—4°C and 7°C was based on the EURL *L. monocytogenes* technical guidance document (EURL Lm 2021). 4°C is a common reference standard for desired cold chain temperatures. Whereas the EURL Guidance recommends 7°C as an appropriate realistic observed storage temperature at manufacturer and retail level in the absence of detailed monitoring data. Preliminary modeling of the maximum growth rates for *L. monocytogenes* at 4°C and 7°C using the online Combase predictive tool (https://combase.errc.ars.usda.gov/) indicated that the maximum growth rate at 7°C is nearly twice that at 4°C, demonstrating the significant difference a small temperature change can make.

### 2.2 Model Fitting of *L. monocytogenes* Growth

The previous work by Myintzaw et al. (2022) only used a modified Gompertz equation to model the growth data. In this study, five growth models were used to estimate the maximum growth rate of *L. monocytogenes* : the Baranyi‐Roberts model, the Gompertz model, a spline model, the Easylinear model, and the Trilinear model. Fitting of the Gompertz, Baranyi‐Roberts, and Trilinear models was done using the DMFit tool available in ComBase (https://www.combase.cc/) (Baranyi and Tamplin 2004). Fitting of the Easylinear and spline models was done using the R 4.3.1 growthrates package (Hall et al. 2014; Kahm et al. 2010), available at https://github.com/tpetzoldt/growthrates/.

The Baranyi‐Roberts model is:

$$y\left(t\right)=y_{0}+\mu_{\max}\times A\left(t\right)-\ln\left(\;1+\frac{e^{\mu_{\max}\times A\left(t\right)}-1}{K-y_{0}}\right)$$

where *y*(*t*) is the log‐transformed microbial count at time *t*, *y*_0_ is the initial log‐transformed microbial count, μ_max_ is the maximum specific growth rate, *K* is the carrying capacity (maximum possible microbial count), and *A*(*t*) is an adjustment function accounting for the lag phase. The adjustment function *A*(*t*) is defined as:

$$A\left(t\right)=t+\frac{1}{\mu_{\max}}\;\ln\left(\frac{e^{-{\mu_{\max}}^{t}}+e^{-h_{0}}-1}{1+e^{-h_{0}}}\right)$$

where *h*_0_ is a parameter that describes the initial physiological state of the microbial population and the length of the lag phase.

The modified Gompertz model is (Zwietering et al. 1990):

$$Y\left(t\right)=Y_{0}+\left(Y_{\max}-Y_{0}\;\right)\;\exp\left\{-\exp\;\left[\frac{{\mu_{\max}}^{e}}{Y_{\max}-Y_{0}}\left(\;\lambda -t\right)+1\right]\right\}$$

where *Y*(*t*) is the log‐transformed microbial count at time, *Y*_0_ is the initial log‐transformed microbial count, *Y*_max_ is the maximum log‐transformed microbial count (i.e., the stationary phase count), *μ*_max_ is the maximum specific growth rate, and *λ* is the lag phase duration.

The Trilinear model is a linear piecewise model that divides the growth curve into three linear segments: lag phase, exponential phase, and stationary phase as follows (Buchanan et al. 1997):

$$\mathrm{Lag}\;\text{phase}:N\left(t\right)=N_{0}+\mu_{1}t$$

$$\text{Exponential phase}:N\left(t\right)=N_{0}+\mu_{2}t$$

$$\text{Stationary phase}:N\left(t\right)=N_{0}+\mu_{3}t$$

where *N*_0_, *N*_1_, and *N*_2_ are the population sizes at the beginning of each phase, and *μ*_1_, *μ*_2_, and *μ*_3_ are the growth rates in each phase.

Spline models use piecewise polynomials to fit data, providing a smooth curve that can represent complex growth patterns. The method is nonparametric because the growth rate is directly estimated from the smoothed data without being restricted to a specific model formula (Suk et al. 2019). The_splines function in the growthrates package of R (Hall et al. 2014) applied a smooth spline function to the data of each group for smoothing spline fitting, and the default smoothness used was 0.5.

The EasyLinear model is a simplified linear model used for describing microbial growth. The growthrates package in R uses the “simple method of growth rates” proposed by Hall et al. 2014 to fit linear models to exponential growth periods. This method fits parts of the linear model to log‐transformed data and attempts to find the maximum growth rate. In the fitting process, a five‐point sliding method is used. The linear model can be expressed as:

$$N\left(t\right)=N_{0}+\mathrm{\mu t}$$

where *N*(*t*) is Ln (population size at time *t*), *N*_0_ is Ln ^(initial population size)^, and *μ*_max_ is the growth rate.

### 2.3 Statistical Analysis

After fitting the growth curves of the isolates using the five models, to ensure the stability of growth data, the coefficient of variation (CV, the ratio of the standard deviation to the mean) was used as a criterion for data screening. The maximum growth rates from the three replicates of each isolate were averaged for each of the five models. The CV of these five average maximum growth rates was then calculated for each isolate. If the CV exceeded 40%, indicating significant variability, the growth curves were visually inspected. If the growth curves showed obvious anomalies, that isolate was discarded from further analysis. Due to difficulties in estimating growth rates for five isolates due to data scatter, only the maximum growth rates of 145 isolates at 4°C and 7°C were subsequently used in this study.

The *L. monocytogenes* isolates were obtained from either clinical sources or food production facilities categorized in several broad categories (Dairy, Meat, Mixed food, Seafood, Vegetable). The Kruskal–Wallis rank sum test was used to detect whether there were significant differences between the isolates from different environments. Subsequently, pairwise comparisons using the Wilcoxon rank sum exact test were used to discern significant differences between pairs of source groups. The statistical analysis was performed in R 4.3.1.

## 3 Results

### 3.1 Model Fitting

**FIGURE 1:**
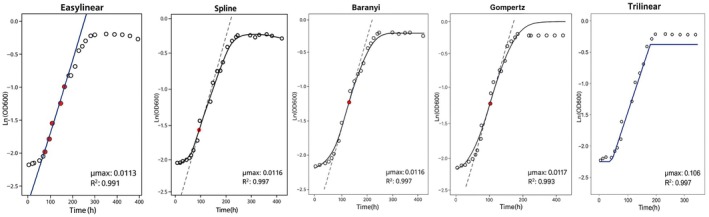
Growth curves and fitted maximum growth rate estimations using the five chosen models ( *L. monocytogenes* isolate 2240, 4°C, replicate 1).

**FIGURE 2:**
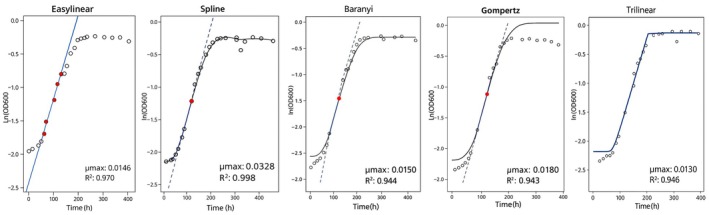
Growth curve and fitted maximum growth rate estimations using the five chosen models ( *L. monocytogenes* isolate 3448, 4°C, replicate 1).

**FIGURE 3:**
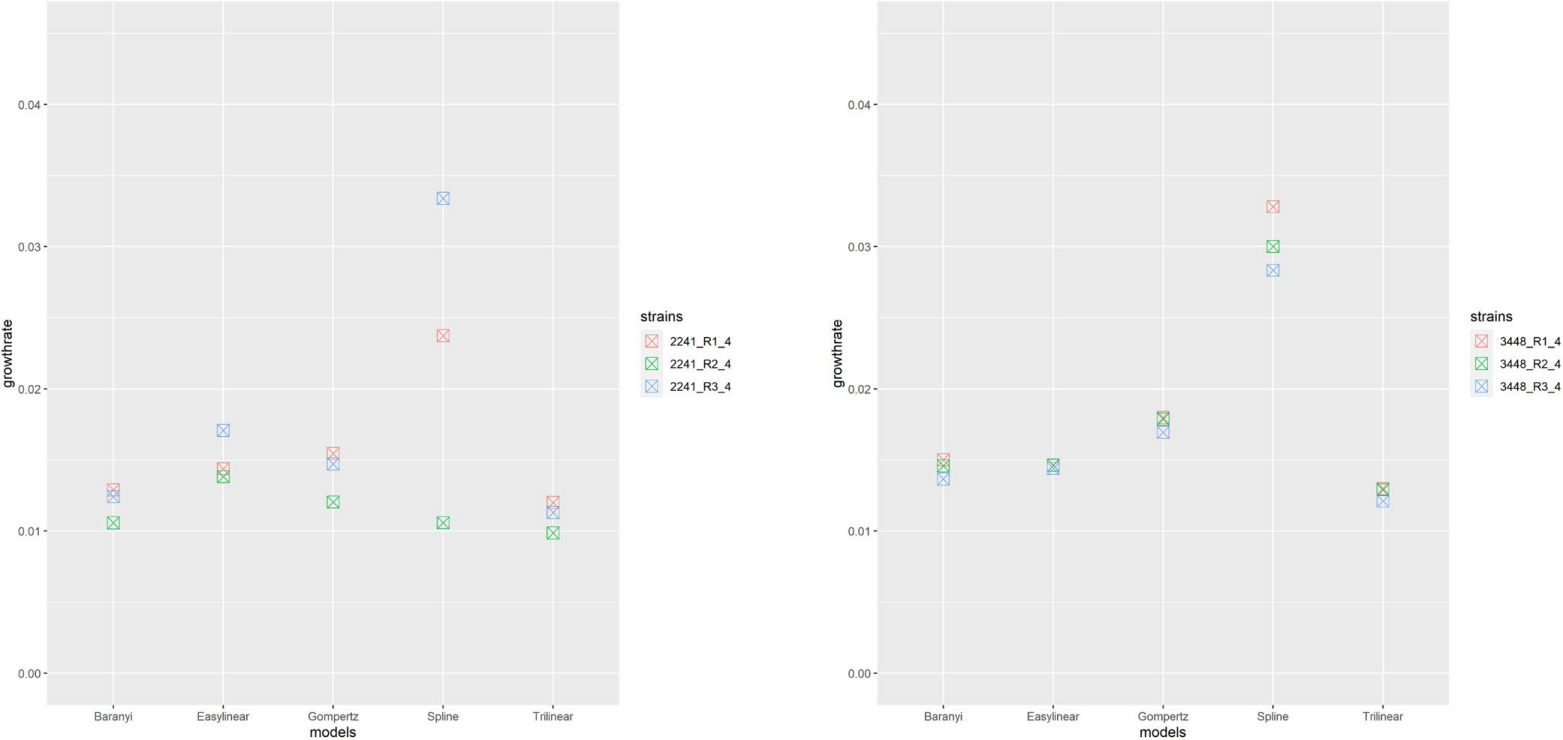
Maximum growth rates of *L. monocytogenes* isolates 2240 and 3448 at 4°C fitted by the five models (three replicates for each isolate).

Figure 1 shows typical growth curves with the five different models superimposed and the tangent line representing the maximum growth rate using the first replicate of isolate 2240 at 4°C as an example. In this example, there was good agreement in the estimated growth rate between the five methods. In contrast, Figure 2 shows that for the first replicate of isolate 3448 at 4°C, the slope of the tangent indicated by the Spline model was notably steeper than that of the other models. Specifically, the maximum growth rate derived from the Spline model was 0.03282 ln (OD_600_)/h, while the growth rates from the other models range between 0.013 and 0.018 ln (OD_600_)/h. Figure 3 shows the maximum growth rates of the two isolates 2240 and 3448 for the three replicates at 4°C. For isolate 2240, the Baranyi, Gompertz, Easylinear, and Trilinear models demonstrated the most consistency between replicates. In contrast, the Spline model showed the largest fluctuations. For isolate 3448, the Spline model's estimate of maximum growth rate was considerably different from the other model for all three replicates.

### 3.2 Maximum Growth Rates

**FIGURE 4:**
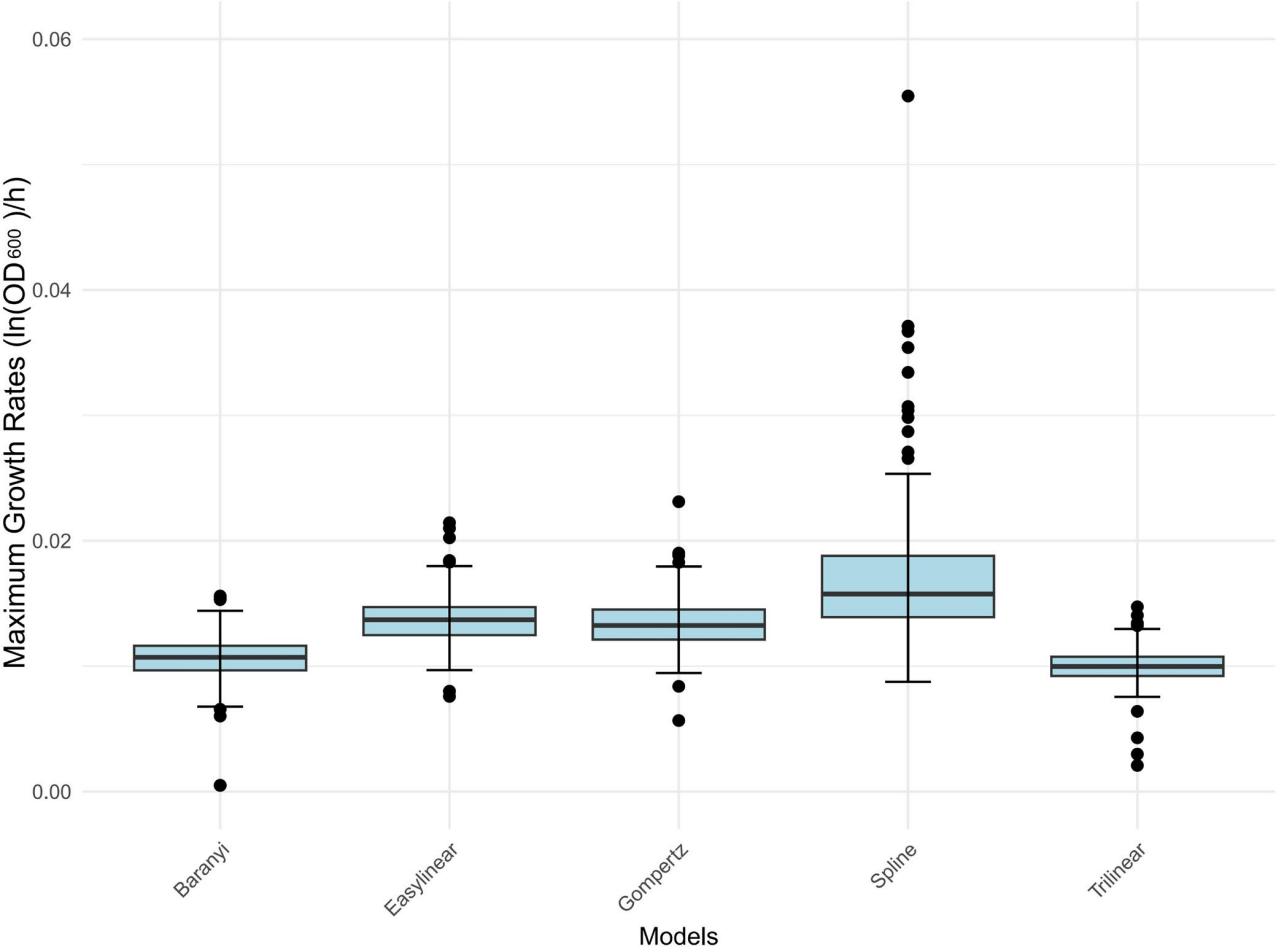
Maximum growth rates for all 145 *L. monocytogenes* isolates at 4°C fitted by five different growth models.

**FIGURE 5:**
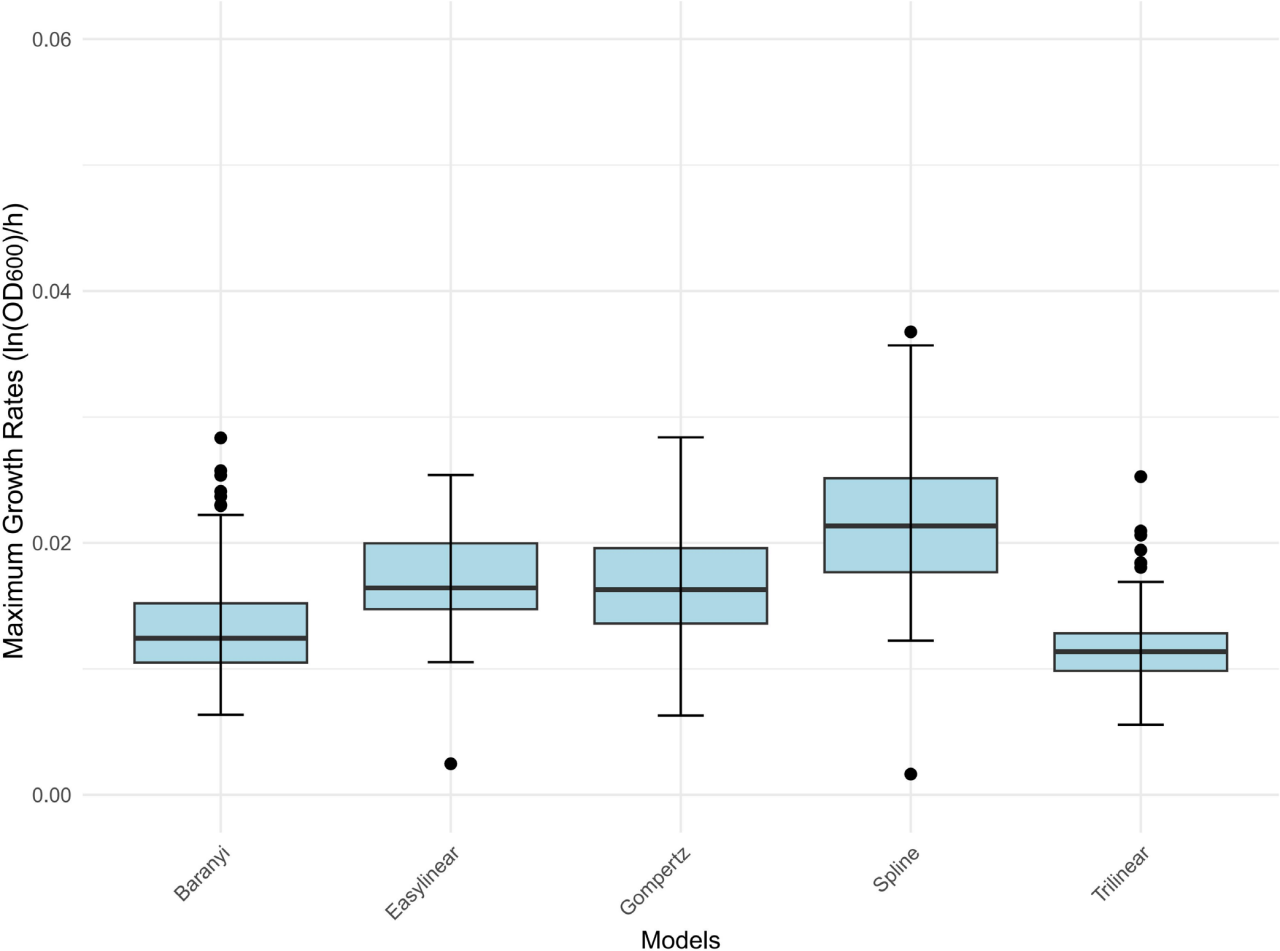
Maximum growth rates for all 145 *L. monocytogenes* isolates at 7°C fitted by five different growth models.

Figures 4 and 5 show the variation in the maximum growth rates for the 145 *L. monocytogenes* isolates obtained using the five models at 4°C and 7°C, respectively. The Spline model showed the highest mean and standard deviation at both temperatures, with a large difference between the maximum and minimum values, indicating significant variability in its fitting ability. The other models had similar means and standard deviations, reflecting a more moderate degree of dispersion. As the spline model was less reliable than the other models, an average maximum growth rate from the four other models—Trilinear, Easylinear, Baranyi, and Gompertz—was used for subsequent analysis of the isolates.

**FIGURE 6:**
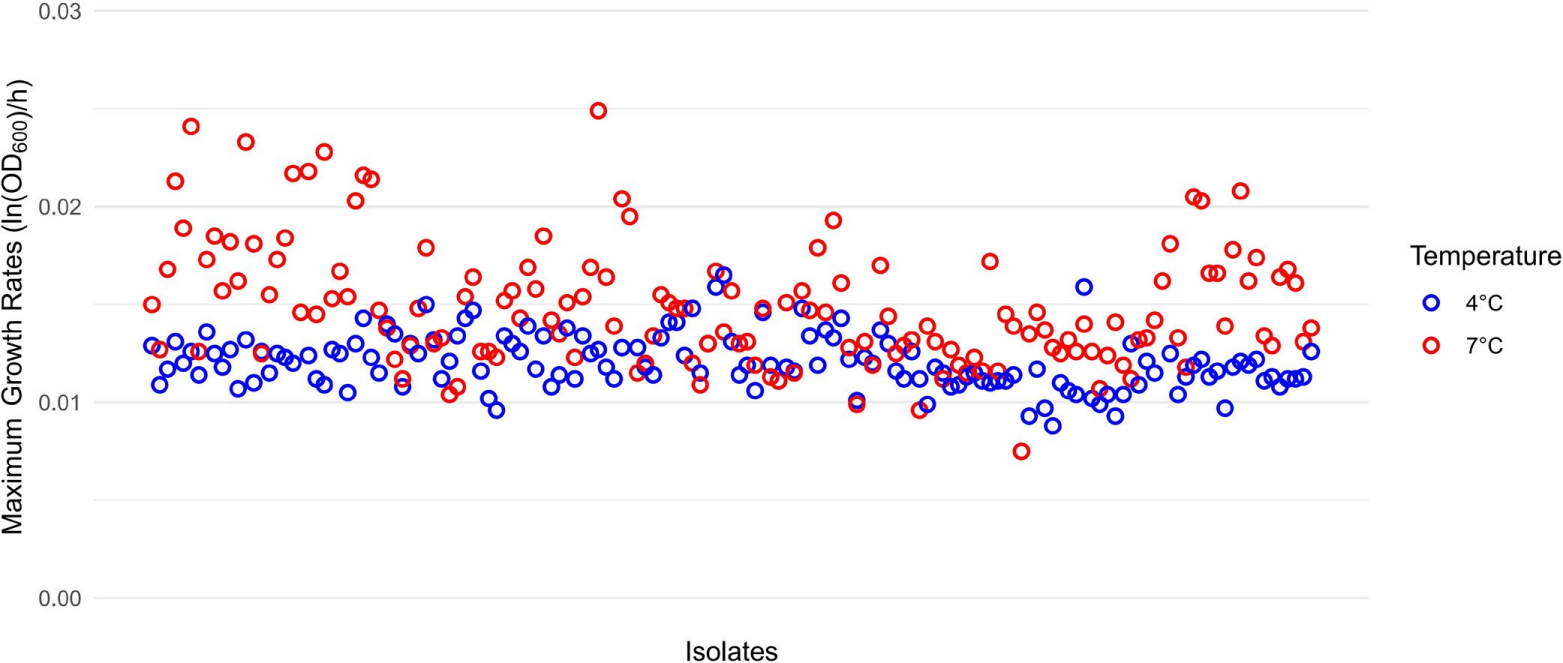
Model‐fitted average maximum growth rates of all *L. monocytogenes* isolates at 4°C and 7°C.

Figure 6 shows the distribution in the calculated average maximum growth rates for all isolates at 4°C and 7°C. The plot shows the considerable variation in maximum growth rates at both temperatures, but particularly at 7°C. It is worth noting that although the maximum growth rate of most isolates at 7°C was higher than that at 4°C, there were still isolates whose estimated maximum growth rate decreased when the temperature increased from 4°C to 7°C.

### 3.3 Impact of Environmental Factors on Growth Rate

**FIGURE 7:**
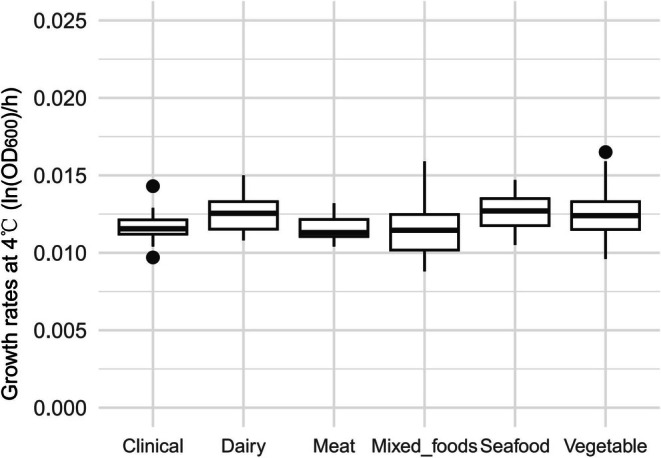
Maximum growth rates for *L. monocytogenes* isolates from different sources at 4°C.

**FIGURE 8:**
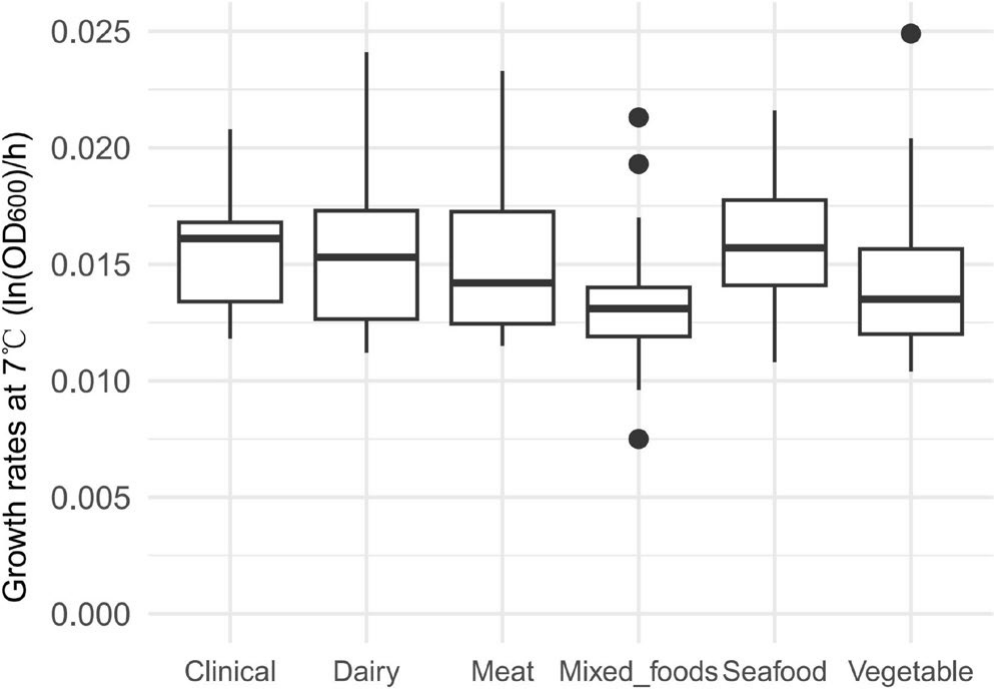
Maximum growth rates for *L. monocytogenes* isolates from different sources at 7°C.

**FIGURE 9:**
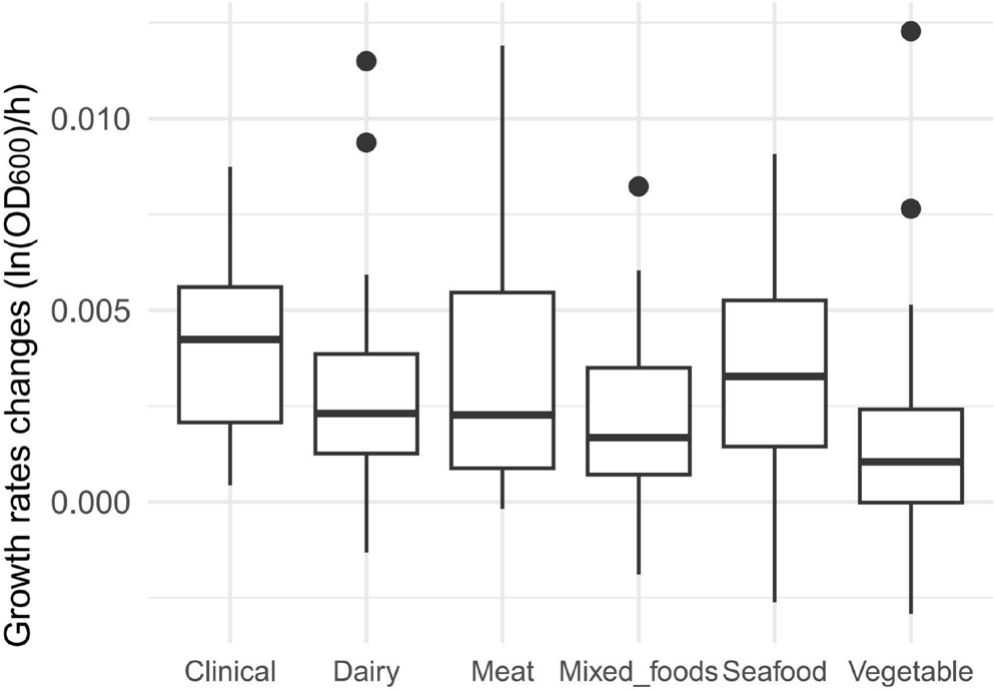
Differences in maximum growth rates for *L. monocytogenes* from different sources between 7°C and 4°C.

Table 1 shows the average maximum growth rates of *L. monocytogenes* isolates recovered from different environments at 4°C and 7°C, as well as the increase in maximum growth rate when the temperature changed from 4°C to 7°C. Figures 7 and 8 are box plots of the growth rates from different environmental at 4°C and 7°C, respectively. There were significant differences depending on the environment in the growth rates at 4°C (*p* < 0.001), 7°C (*p* < 0.01), and in the difference in growth rates after the temperature was increased (*p* < 0.01). According to the pairwise comparisons using the Wilcoxon rank sum exact test, at 4°C, the growth rate of *L. monocytogenes* isolates collected from seafood processing facilities was significantly higher than that of isolates collected from meat facilities (*p* < 0.01). At 7°C, the growth rate of *L. monocytogenes* isolates collected from seafood facilities was significantly higher than that of isolates collected from mixed food facilities (*p* < 0.01). However, the magnitude of the overall differences was relatively small. When the temperature increased from 4°C to 7°C, the change in growth rate of *L. monocytogenes* isolated from vegetable processing facilities was higher than that of *L. monocytogenes* isolates recovered from clinical sources (*p* < 0.01, Figure 9).

**TABLE 1:** Average maximum growth rates of *L. monocytogenes* isolates collected from different environments.

Source	Number of isolates	Average maximum growth rates (ln (OD_600_)/h)		
		4°C, mean (SD)	7°C, mean (SD)	Change from 4°C to 7°C, mean (SD)
Clinical	24	0.0117 (0.0009)^ab^	0.0157 (0.0026)^pq^	0.0041 (0.0024)^x^
Dairy	14	0.0125 (0.0012)^ab^	0.0158 (0.0037)^pq^	0.0033 (0.0035)^xy^
Meat	23	0.0116 (0.0007)^a^	0.0152 (0.0036)^pq^	0.0036 (0.0034)^xy^
Mixed food	28	0.0115 (0.0017)^ab^	0.0134 (0.0025)^p^	0.0020 (0.0023)^xy^
Seafood	27	0.0127 (0.0012)^b^	0.0160 (0.0027)^q^	0.0033 (0.0028)^xy^
Vegetable	29	0.0126 (0.0017)^ab^	0.0142 (0.0031)^pq^	0.0016 (0.0031)^y^
All	145	0.0121 (0.0014)	0.0149 (0.0031)	0.0032 (0.0036)

*Note:* Letters indicate significant differences (*p* < 0.05) within this dataset.

### 3.4 Comparison of Maximum Growth Rates Using Turbidity and Plate Count

**FIGURE 10:**
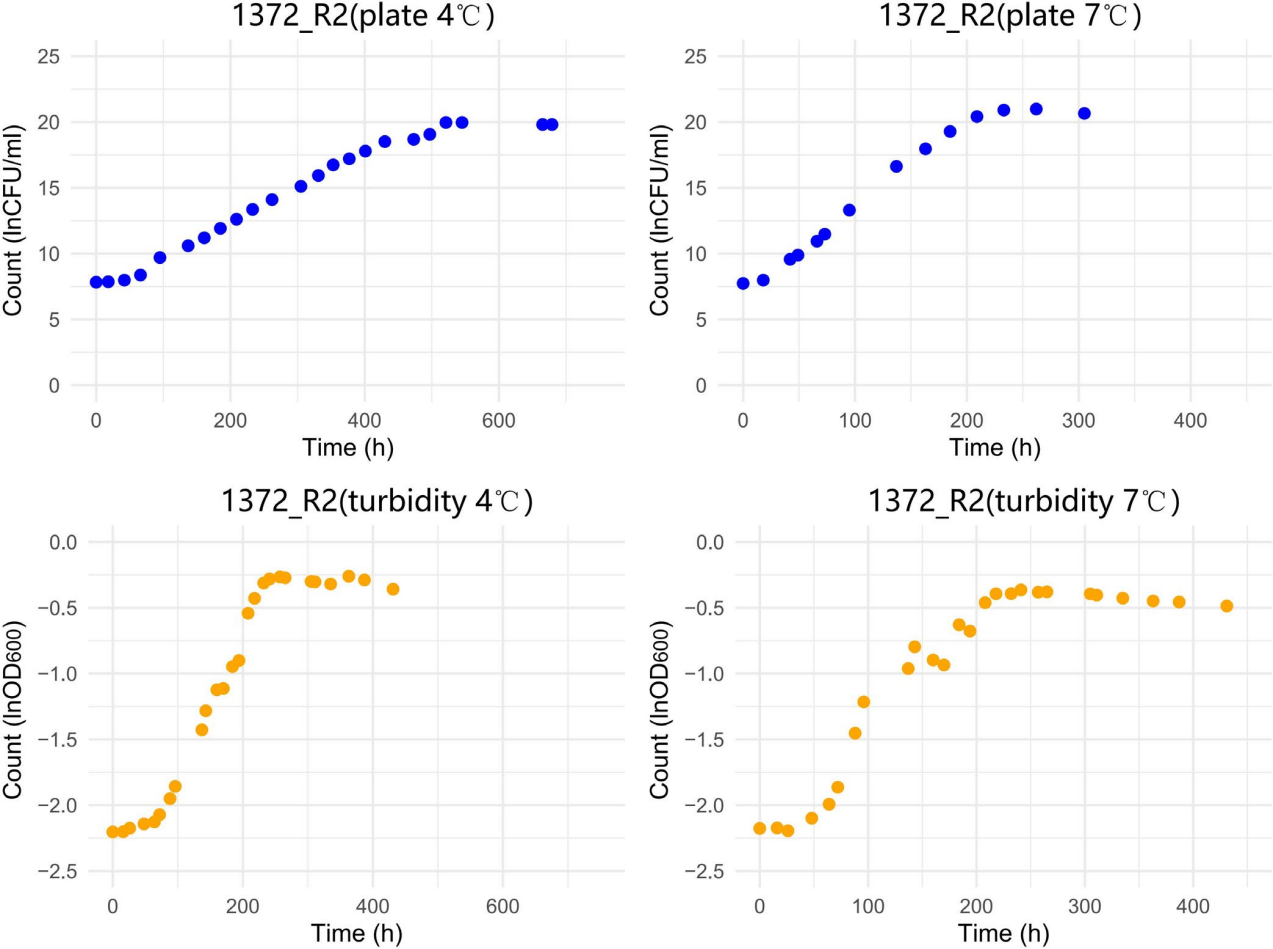
Growth curves of *L. monocytogenes* F2365 in plate and liquid culture at 4°C and 7°C.

For a small number of isolates, the growth of *L. monocytogenes* strains was monitored at 4°C and 7°C in both turbidity and plate count, with bacterial counts measured at different time points. Using the 1372 strain as an example (Figure 10), a clear growth phase was observed in both environments. However, the growth phase on plates commenced earlier and exhibited a steeper increase, whereas by turbidity, although a growth phase was also evident, the increase was more gradual and extended over a longer period. As the temperature was reduced to 4°C, the maximum growth rate in both environments slowed, and the growth phase was prolonged. Similar trends were observed for other strains (data not shown).

## 4 Discussion

This study assessed the growth modeling of *L. monocytogenes* across various temperatures and environmental conditions to evaluate its growth kinetics using five different mathematical models. The results highlight variability in growth rate estimates depending on the model used. The Spline model, for example, produced significantly higher and more variable maximum growth rates, likely due to overfitting (Schuster et al. 2022). Since spline fitting directly estimates slopes from local curve segments, it can track random fluctuations in noisy growth data and thus yield inflated or unstable estimates of the maximum growth rate. In contrast, nonlinear models like Baranyi and Trilinear have been validated as highly accurate (López et al. 2004), largely because their trajectories are constrained by biologically plausible growth functions, which reduces the influence of noise. Similarly, simpler linear models—such as Easylinear—also demonstrated stability, providing growth rate estimates comparable to those of nonlinear approaches.

In addition to model choice, effects of environmental factors on growth were also observed. This study found that *L. monocytogenes* isolates from seafood‐processing facilities exhibited slightly higher growth rates than those from meat‐processing environments. This aligns with findings from an EU‐wide survey (EFSA 2013), which reported that *L. monocytogenes* prevalence was highest in smoked fish (10.3%) compared to heat‐treated meat (2.07%) and cheese (0.47%). The proportion of samples exceeding the regulatory limit of 100 CFU/g was also greatest in fish (1.7%). The elevated growth potential observed in seafood isolates may explain why seafood products present a higher relative risk. Nevertheless, although these source‐level differences were statistically significant, their magnitude was small (Table 1), indicating limited practical relevance compared with the dominant effect of temperature. From a risk assessment perspective, this suggests that temperature management should take precedence, with source‐related variation treated as secondary.

Building on this, our results illustrate how even small changes in temperature can markedly alter growth dynamics. At 4°C, growth remains slow, but at 7°C, doubling times shorten, increasing the likelihood of exceeding safety limits before the product's expiration date. These results align with Codex guidelines, which recommend maintaining refrigerated ready‐to‐eat foods at ≤ 4°C to limit *L. monocytogenes* proliferation (Codex Alimentarius Commission 2007). Our models reinforce that a mere 3°C difference can shift the risk profile from acceptable to hazardous, highlighting the importance of strict cold chain management. Interestingly, a small number of isolates showed lower growth rates at 7°C than at 4°C. This may reflect strain‐specific cold adaptation or physiological variability, such as differences in membrane composition or stress responses near suboptimal temperatures. Previous studies have also shown that cold tolerance varies among genetic lineages of *L. monocytogenes* and involves distinct adaptive mechanisms (Hingston et al. 2017; Cordero et al. 2016), suggesting that further genetic and physiological analyses are needed to clarify these differences.

The findings of this study also raise questions regarding the practical use of predictive modeling. The comparison of models demonstrated that growth rate estimates may vary depending on the fitting approach, which suggests that the degree of precision and flexibility required for risk assessment could differ across contexts. While the growthrates package facilitates detailed analyses (Petzoldt 2019), ComBase DMFit offers a more accessible interface for routine applications. Future work could explore how such approaches might be integrated or adapted to accommodate data with greater variability, for example under fluctuating storage conditions, thereby bringing predictive models closer to the complexity of real cold‐chain systems.

## 5 Conclusion

In conclusion, this study examined the growth dynamics of *L. monocytogenes* at low temperatures (4°C and 7°C), utilizing several growth models to assess their reliability. The Baranyi, Gompertz, Trilinear, and Easylinear models provided consistent and reliable fits, while the Spline model showed higher variability and was less dependable. There was on occasion significant variability in the estimates of growth rates observed, depending on the model chosen. The environmental source of the isolates had a small influence on growth rate. This finding highlights the need for further work to consider both genetic and environmental factors in managing *L. monocytogenes* risks in the cold chain. This study emphasizes the need to evaluate several models when doing predictive microbiological analysis. The work also highlights the need, when considering a large number of growth curves, to do appropriate data cleaning, particularly when the growth data is obtained from turbidity measurements. Using multiple methods of estimating growth rates helps in this data cleaning step.

## Data Availability

The data that support the findings of this study are available from the corresponding author upon reasonable request.
